# *Short Communication*: Interaction of Nerve Growth Factor (NGF) and Vascular Endothelial Growth Factor (VEGF) in Healthy Individuals

**DOI:** 10.1155/2019/7510315

**Published:** 2019-11-11

**Authors:** Adrian Groh, Kirsten Jahn, Michael Gröschl, Thomas Hillemacher, Johannes Kornhuber, Stefan Bleich, Helge Frieling, Annemarie Heberlein

**Affiliations:** ^1^Department of Psychiatry, Social Psychiatry and Psychotherapy, Center for Addiction Research, Medical School Hanover, Germany; ^2^Celerion Switzerland AG, Switzerland; ^3^Paracelsus Medical University Nuremberg, Germany; ^4^Department of Psychiatry and Psychotherapy, Friedrich-Alexander University Erlangen-Nürnberg (FAU), Germany

## Abstract

NGF and VEGF are known to be involved in different psychiatric diseases. In order to verify hints from basic research that both neurotrophines interact with each other, serum levels of NGF and VEGF were measured in a cohort of 33 healthy individuals and correlated. NGF level was 126.30 pg/mL (±155.43), and VEGF level was 57.28 pg/mL (±44.48). Both factors were significantly correlated, confirming their interaction and legitimising the usage of their respective ratio (0.8 (±0.42)) as a less varying additional marker in prospective studies.

## 1. Introduction

Nerve growth factor (NGF) and vascular endothelial growth factor (VEGF) are important signal molecules and can both exert multiple effects: NGF is able to regulate growth, differentiation, and survival of neurons [1]. Furthermore, it is known to be involved in the regulation of inflammation and to interact with various cells of the immune system [2]. Another effect of NGF is the promotion of angiogenesis in tissues like the skin, the skeletal muscle, the cornea, and the central nervous system [3]. In the CNS, it is mainly produced in the cortex, hippocampus, and pituitary gland as well as in the basal ganglia, thalamus, spinal cord, and retina. Peripherally, also, immune hematopoietic cells produce NGF [4] and adipose tissue cells [5].

VEGF is known for its key role in angiogenesis (for review, see [6]). Various study results show that VEGF promotes vascularisation and also increases vascular permeability [7]. Besides affecting angiogenesis and vascular permeability, VEGF acts as a neuroprotective neuropeptide in the case of serum deprivation [8] and glutamatergic neurotoxicity [9]. It can be produced by various cell types like macrophages, platelets, keratinocytes, and renal mesangial cells and in pathological conditions also by tumor cells [10].

There is increasing evidence that disturbances in VEGF, NGF, and other growth factors play a major role in neurologic and psychiatric illnesses like major depression, dementia, schizophrenia, and alcohol use disorders [11–13]. For instance, VEGF and NGF were shown to be decreased in patient's serum [14, 15]. Therefore, these and other growth factors could even serve as clinical biomarkers in psychiatric disorders in order to facilitate diagnosis and estimation of prognosis, as long as patients are not codiagnosed with cancer and infectious or vascular diseases. For a comprehensive review, see Lazarovici et al. [16] resp., Galvas-Contreras et al. [17].

Lately, both neuropeptides have been reported to interact with each other. Middeke et al. for example could show *in vitro* and *in vivo* in a mouse model that NGF induces VEGF expression [18]. Angiogenic effects of NGF are discussed to be mediated by an increase of VEGF expression [19].

Indeed, on a molecular level, NGF has an impact in part of signal cascades that are activated by VEGF: the Ras/ERK and PI3K/Akt pathways. Combined with the results reported above, this might point towards a modulating role of NGF on VEGF [20, 21].

In order to find hints in living human objects for this correlation indicated by the mentioned preclinical studies, we measured and correlated VEGF and NGF serum levels in healthy volunteers and calculated their ratio. Additionally, it is often difficult to draw conclusions from highly varying absolute values which are observable in control groups of most studies [12, 22, 23] and which are not relatable to demographic factors like age [24]. Therefore, in case of a clear correlation between both factors, the ratio of both factors could mathematically help to overcome the interindividual variations. In this case, the ratio could prospectively be used as a less variant marker, facilitating assessment of findings in individual patients as well as comparison between different groups.

## 2. Materials and Methods

The present study was part of a large Neuroendocrinology and Neurogenetics prospective research arrangement (NENA) that was approved by the local Ethics Committee of the University of Erlangen-Nuremberg. The investigation was conducted in accordance with the Declaration of Helsinki of 1975 (revised in 1983). Each participant gave written informed consent.

In total, we investigated serum levels of VEGF and NGF in 33 healthy individuals (20 female, 13 male). Subjects were negative for psychiatric diagnoses, existence of severe somatic illnesses like cancer, ischemia, and degenerative or infectious diseases as well as substance abuse apart from nicotine. All the subjects underwent a detailed physical examination, routine laboratory testing, and urine drug screening. Screening for alcohol dependence and abuse was done by use of the CAGE questionnaire and the alcohol use disorder identification test—(AUDIT-C). Data regarding affective symptoms were collected by the Beck's Depression Inventory (BDI) and the State and Trait Anxiety Inventory (STAI). All test persons were negative for alcohol abuse, alcohol dependence, or other mental diseases according to ICD-10.

For further analysis, blood samples after fasting were taken between 8 and 10 a.m. Serum samples were kept at room temperature for 45 minutes and were then centrifuged at 1400 g for 10 min. at 4°C. The supernatant was stored at -80°C till further analysis.

The human VEGF- and the human *β*-NGF-levels were assessed using the DuoSet enzyme-linked immunosorbent assay (ELISA) Development System (DY293 B, DY256, R&D Systems, Wiesbaden-Nordenstadt, Germany). All the assays were performed according to the manufacturer's description. Standard curves revealed a detection range of 31.2-2000 pg/mL for VEGF and 62.5 to 4000 pg/mL for NGF, yielding a lower limit of determination of 45 pg/mL. The intra-assay coefficients of variation were 5.8 and 7.2%, respectively. The sample concentrations in each plate were calculated according to standard curves and dilution factors.

Additional data like age and body mass index (BMI) were obtained in a structured interview.

### 2.1. Statistical Analysis

The hypothesis of normal distribution was rejected by means of the Kolmogorov-Smirnov test (K-S test) for absolute values of NGF and VEGF. Therefore, nonparametric tests were used for correlation and regression. Correlations were calculated by the Spearman's correlation coefficient. Influence of NGF on VEGF was calculated by linear regression analysis setting with VEGF as dependent variables. VEGF/NGF ratios showed a normal distribution. Data were analysed employing SPSS Statistics 26 and GraphPad Prism™ 8 (GraphPad Software Inc., San Diego, CA).

## 3. Results

The cohort consisted of 33 subjects (mean age = 34.91; 13 males, 20 females). 5 of them were smokers, and the mean BMI was 23.85. Demographic data of the test cohort is also given in Table 1.

**Figure 1:**
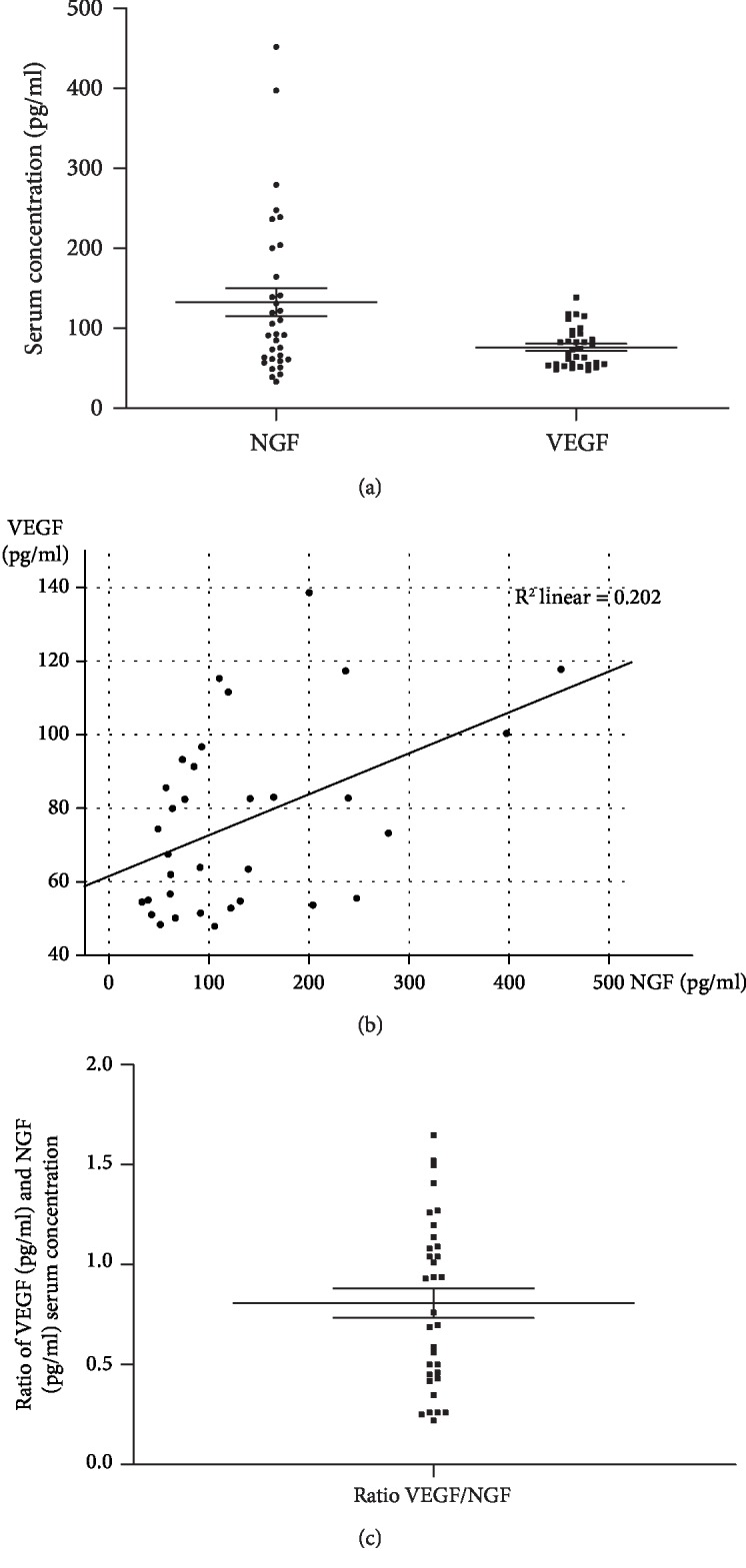
(a) The serum concentration of NGF and VEGF in pg/mL. (b) The correlation of NGF and VEGF values (pg/mL) as indicated by the regression line. (c) The ratio of the NEGF and VEGF serum concentration.

NGF showed a concentration of 132.8 pg/mL (±100.9) and VEGF a concentration of 76.2 pg/mL (±24.5) serum (Figure 1(a)). Neither NGF nor VEGF levels showed age-, gender-, or BMI-related changes. Serum levels of NGF and VEGF were not normally distributed (K-S test) but significantly correlated with each other in this cohort of healthy persons (Spearman's rho = 0.407, *p* = 0.019). Linear regression analysis further confirmed the association between VEGF and NGF serum levels (*B* = 1.85 ± 0.66; *β* = 0.449, *p* = 0.009; *F* = 7.84) (Figure 1(b)). The VEGF/NGF ratio was 0.81 ± 0.42 (Figure 1(c)) and showed a normal distribution (K-S test).

## 4. Discussion

Nowadays, more and more efforts are made to identify biomarkers in almost all fields of medicine in order to achieve more tools for earlier and more precise diagnosis and to facilitate therapeutic decisions as well as the estimation of prognoses [25]. Especially, in psychiatric diseases, identification of the right individual therapy strategy is often time-consuming and therefore implies a great burden for patients. Due to the fact that growth factors (besides neurotransmitters) have a big impact in the pathogenesis of most psychiatric disorders and due to their good accessibility via blood withdrawal, they became more and more interesting as biomarkers in the whole field of psychiatry [17].

Particularly, disturbances of NGF and VEGF have been described in most psychiatric diseases [11, 12] and could therefore be especially suitable as indicators.

Nevertheless, absolute “normal” serum standard values for NGF and VEGF are not easy to be defined. For instance, there are large variations between control groups of different studies and even within each study, the control groups show high SDs or IQRs. Liu et al. for example showed serum NGF levels of 1.9 pg/mL (±0.38) (SD) in their control group [23] whereas Jockers-Scherubl et al. measured 42.1 pg/mL (±68.0) (SD) [12]. Bansal et al. showed VEGF levels of 171.6 pg/mL (IQR 91.99-280.8) [22]. In the present study, absolute values for NGF (mean value 132.77 pg/mL ±100) also varied between 32.96 (min.) and 452.03 (max.) pg/mL, and values for VEGF (76.19 pg/mL ±24.5) showed a range between 47.91 (min.) and 138.59 (max.) pg/mL. In both cases, values were not normally distributed. The broad range of “normal” serum values could be explained either by physiological individual variations or by differences in laboratory measuring procedures but were not relatable to demographic factors like age or gender. In contrast, some studies suggest an age-related decrease of VEGF [26, 27] and NGF expression [28]. However, these studies mostly refer to biopsies of single organs from rats and cell culture experiments. Furthermore, our results are in line with reports investigating VEGF or NGF serum levels in humans, which also did not reveal any gender and age dependency [24, 29]. In literature, many pathological conditions showed significant differences compared to respective control groups, but absolute values were overlapping that much that it would be difficult to draw conclusions from individual values for a patient [12, 22].

As basic research suggests an interdependency between NGF and VEGF [20, 21], serum levels were correlated in this cohort of healthy objects. Indeed, the correlation analysis (Spearman Rho) confirmed a weak but significant association of both factors (0.407, *p* < 0.05). Therefore, it made sense to calculate their ratio in order to check if this could be an additional marker with more consistency than the absolute values. This could help to correct for interindividual variability and differences in measuring procedures of different laboratories. Therefore, it could make research findings more comparable and facilitate conclusions for individual patients. Mean value of the VEGF/NGF ratios was 0.8 ± 0.42 (with a range from 0.22 to 1.65) and showed a normal and much more even distribution of values, implicating higher stability of the ratio as a marker compared to absolute values. Therefore, the ratio could be a helpful tool to correct for high variation of individual NGF and VEGF values. In our study, the NGF level was 126.30 pg/mL (±155.43) which is a bit higher than in the cited studies above [12, 23], and the VEGF level was 57.28 pg/mL (±44.48), which is lower [22]. Anyway, our values are still in the range of the other studies, and none of these studies measured both factors in the same individuals. Our data indicate that the absolute NGF level is probably higher than the absolute VEGF level and their ratio is about 0.8.

Limiting factors that should be mentioned are the relatively low *n* (number) (although correlation analyses showed no age and gender specifics) and the assessment of diseases only via questionnaires.

In summary, our data suggest that the additional use of the VEGF/NGF ratio could be a useful approach to facilitate interpretation of individual VEGF and NGF serum levels as well as comparison of groups. This concept should be further verified for different pathologies in future studies.

To our knowledge, this study is the first that investigates a possible correlation of both factors in healthy human subjects, thereby supporting respective hints from animal models that were indicating an interaction between VEGF and NGF expression via certain signal cascades.

## Figures and Tables

**Table 1:** The demographic data of the individuals.

	*n*	Mean value	SD	Min/max
Age (years)	33	34.91	16.17	21/73
Gender (male/female)	13/20			
Smoking (no/yes)	28/5			
BMI (kg/m^2)^	33	23.85	3.17	17.53/31.70

## Data Availability

The availability of the underlying data related to the Submission is give.
